# Salicylate •Phenanthroline copper (II) complex induces apoptosis in triple-negative breast cancer cells

**DOI:** 10.18632/oncotarget.16161

**Published:** 2017-03-13

**Authors:** Limei Fan, Muyou Tian, Yunyi Liu, Ying Deng, Zhengkai Liao, Jinhua Xu

**Affiliations:** ^1^ School of Medicine, Jianghan University, Wuhan, Hubei 430056, China; ^2^ Key Laboratory of Optoelectronic Chemical Materials and Devices, Ministry of Education, Jianghan University, Wuhan, Hubei 430056, China; ^3^ Department of Radiation and Medical Oncology, Zhongnan Hospital, Wuhan University, Wuhan, Hubei 430071, China; ^4^ Hubei Key Laboratory of Tumor Biological Behavior, Wuhan University, Wuhan, Hubei 430071, China

**Keywords:** TNBC, Cu(sal)(phen), cell growth, apoptosis, anti-apoptotic protein

## Abstract

In this study, we investigated anti-tumor activity and associated molecular mechanism of action of Salicylate ●Phenanthroline Copper (II) Complex in triple-negative breast cancer. Salicylate ●Phenanthroline Copper (II) Complex inhibited the growth of four breast cancer cell lines (MCF-7, T47D, MDA-MB-231 and BT-20) and induced apoptosis in a concentration-dependent manner. The effect was more profound in MDA-MB-231 and BT-20 triple-negative breast cancer cell lines. Western blot showed that the expression of the apoptosis-related protein Bcl-2, Bcl-xl and survivin was significantly reduced in MDA-MB-231 after treatment with Salicylate ●Phenanthroline Copper (II) Complex. *In vivo*, Salicylate ●Phenanthroline Copper (II) Complex administration significantly attenuated tumor growth of MDA-MB-231 xenografts, and the expression levels of Bcl-2, Bcl-xL and survivin were reduced as measured by immunohistochemical staining. These data suggest that Salicylate ●Phenanthroline Copper (II) Complex is a promising novel therapeutic drug for triple-negative breast cancer and warrants further study.

## INTRODUCTION

Triple-negative breast cancer (TNBC) accounts for 10% to 20% of all breast cancer cases. TNBC is highly aggressive and usually associated with poor prognosis. Unlike other types of breast cancer, TNBC lacks effective targeted therapies since it lacks estrogen receptor, progesterone receptor and HER-2 [1]. Despite significant progress in recent years with the development of potential novel drugs such as PARP inhibitors and bevacizumab, no targeted therapy are currently available for use in the metastatic setting [2, 3]. Chemotherapy is the standard therapy for TNBC patients [4], and neoadjuvant chemotherapy is often used for treatment of early-stage TNBC, which reduces tumor stage and increases the number of patients suitable for breast-conserving surgery [5]. Although many TNBC patients experience remission, the overall survival (OS) rate is still lower compared to non-TNBC patients [2]. TNBC recurrence typically peaks within three years after treatment, and most patients do not survive beyond five years, although the gap between TNBC and non-TNBC survival decreases after ten years [1, 6]. When pathologic complete response (pCR) was not achieved in TNBC patients after neoadjuvant chemotherapy, a significantly poorer OS was observed compared to non-TNBC patients with residual disease [7]. Compared with non-platinum-based chemotherapy, platinum-based agents can significantly improve the pCR rate [8, 9], although a benefit in OS remains to be seen [10]. Importantly, although most TNBC tumors respond well to chemotherapy initially, they often develop drug resistance [11]. Therefore, there is an urgent need to develop new therapeutic agents for TNBC.

Tumors are characterized with uncontrolled proliferation and a suppression of apoptosis, thus targeting those processes may provide effective therapies [12, 13]. The Bcl-2 family of proteins, including anti-apoptosis factors Bcl-xL and Bcl-2 and pro-apoptotic factors Bax, Bik, and Bad, are key apoptosis regulators [14]. Additionally, survivin is a member of the inhibitor of apoptosis proteins (IAP) family that inhibits apoptosis [15]. Both Bcl-2 family proteins and survivin are overexpressed in many types of human cancers, including breast cancer [16, 17]. Survivin, in particular, is a promising candidate for targeted cancer therapy, as its expression is associated with poor clinical outcome, more aggressive clinicopathologic features, and resistance to radiation and chemotherapy [18].

Salicylic acid and salicylate derivative have been widely used in anti-inflammatory and anti-cancer therapy [19, 20]. It has been reported that copper (II) complexes of salicylic acid have similar anti-oxidative and anti-tumor activities as superoxide dismutase (SOD) [21]. Specifically, these compounds inhibit the growth of tumor cells, and Sorenson *et al*., showed that copper salicylic acid had anti-tumor potential in animal models. Treatment led to reduced tumor growth and metastasis, induced tumor cell differentiation, and increased host survival [22, 23]. Other studies have found that copper salicylic acid chelated with phenanthroline increased its toxicity in tumor cells [24]. Cisplatin-sensitive cell lines, such as breast cancer cells (MCF-7), prostate cancer cells (DU-145), and colon cancer cells (HT-29), and cisplatin-resistant cell lines, such as ovarian cancer cells (SK-OV-3), were all sensitive to low doses of copper salicylate phenanthroline complexes [21]. Despite these promising anti-cancer effects of Cu(sal)(phen), its mechanism of action have not been defined.

In this study, we analyzed the effect of Cu(sal)(phen) on breast cancer cell growth *in vitro* and anti-tumor activity *in vivo*. We found that Cu(sal)(phen) efficiently induces apoptosis of TNBC cells through down-regulation of anti-apoptosis proteins in these cells *in vitro* and *in vivo*. Thus, our results suggest that Cu(sal)(phen) is a promising novel therapeutic agent for TNBC.

## RESULTS

### Cu(sal)(phen) inhibits the growth of breast cancer cells

**Figure 1:**
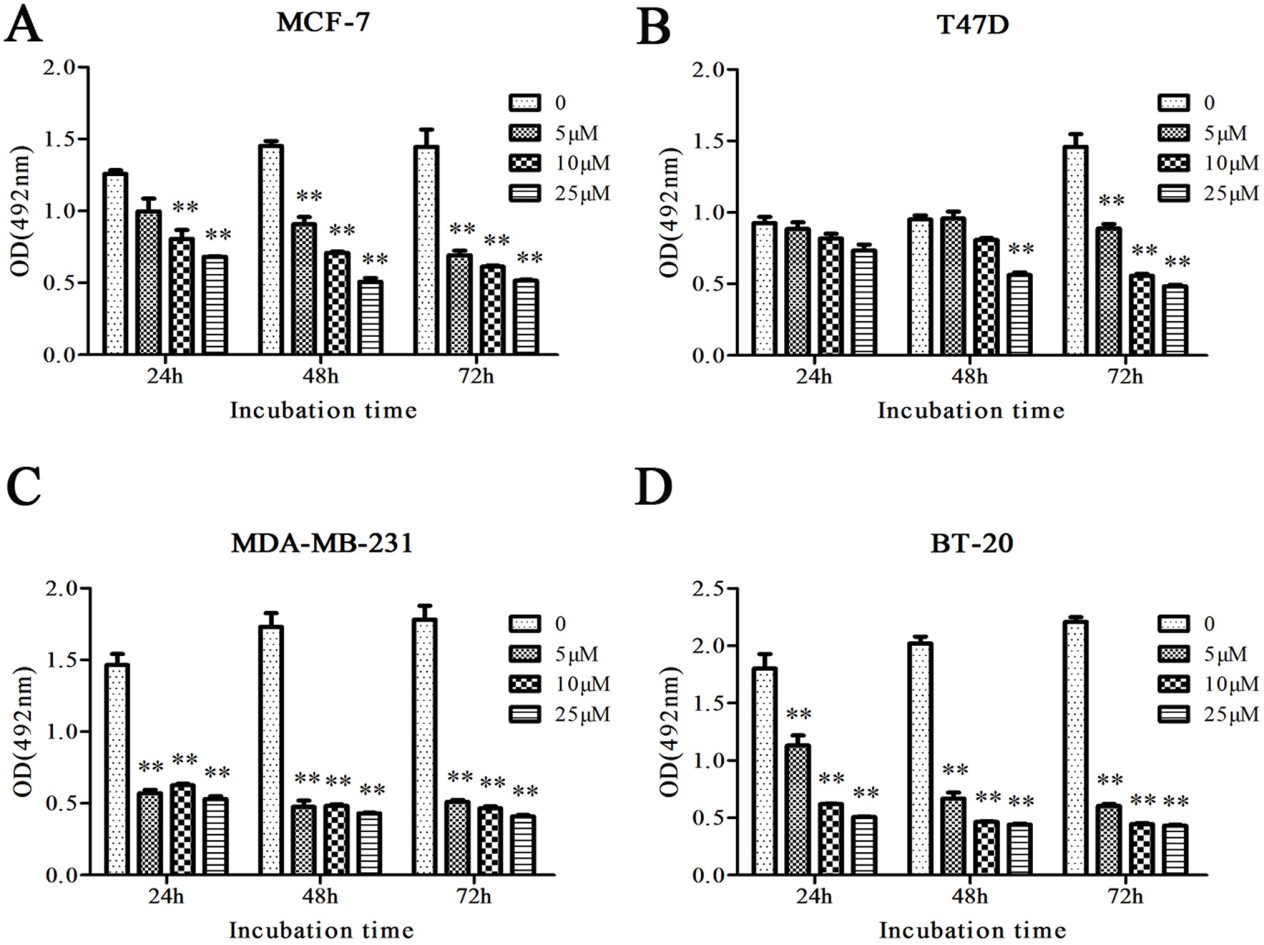
Cu(sal)(phen) inhibits growth of breast cancer cells MCF-7(**A**), T47D(**B**), MDA-MB-231(**C**) and BT-20(**D**) were treated with 0–25 μM of Cu(sal)(phen), and cell growth was measured with the MTS assay at indicated time periods. Results are represented as the average of two independent experiments with triplicates. Bar represents mean ± SD. **p < 0.01.

To determine the effect of Cu(sal)(phen) on growth of breast cancer cells, four breast cancer cell lines (MCF-7, T47D, MDA-MB-231 and BT-20) were treated with increasing concentrations of Cu(sal)(phen) (5–25 μM), and cell growth was assessed by MTS assay. As shown in Figure 1, the growth of all cells was significantly inhibited by Cu(sal)(phen) treatment compared to the control group (*p* < 0.01, Figure 1). There was a dose-dependent effect of the compound; the extent of inhibition was positively correlated with increasing Cu(sal)(phen) concentration. When the Cu(sal)(phen) concentration reached 25 μM, cell growth was almost completely inhibited. In addition, the effect was more profound in MDA-MB-231 and BT-20 TNBC cell lines compared with MCF-7 and T47D non-TNBC cell lines (Figure 1).

### Cu(sal)(phen) induces apoptosis of breast cancer cells

**Figure 2:**
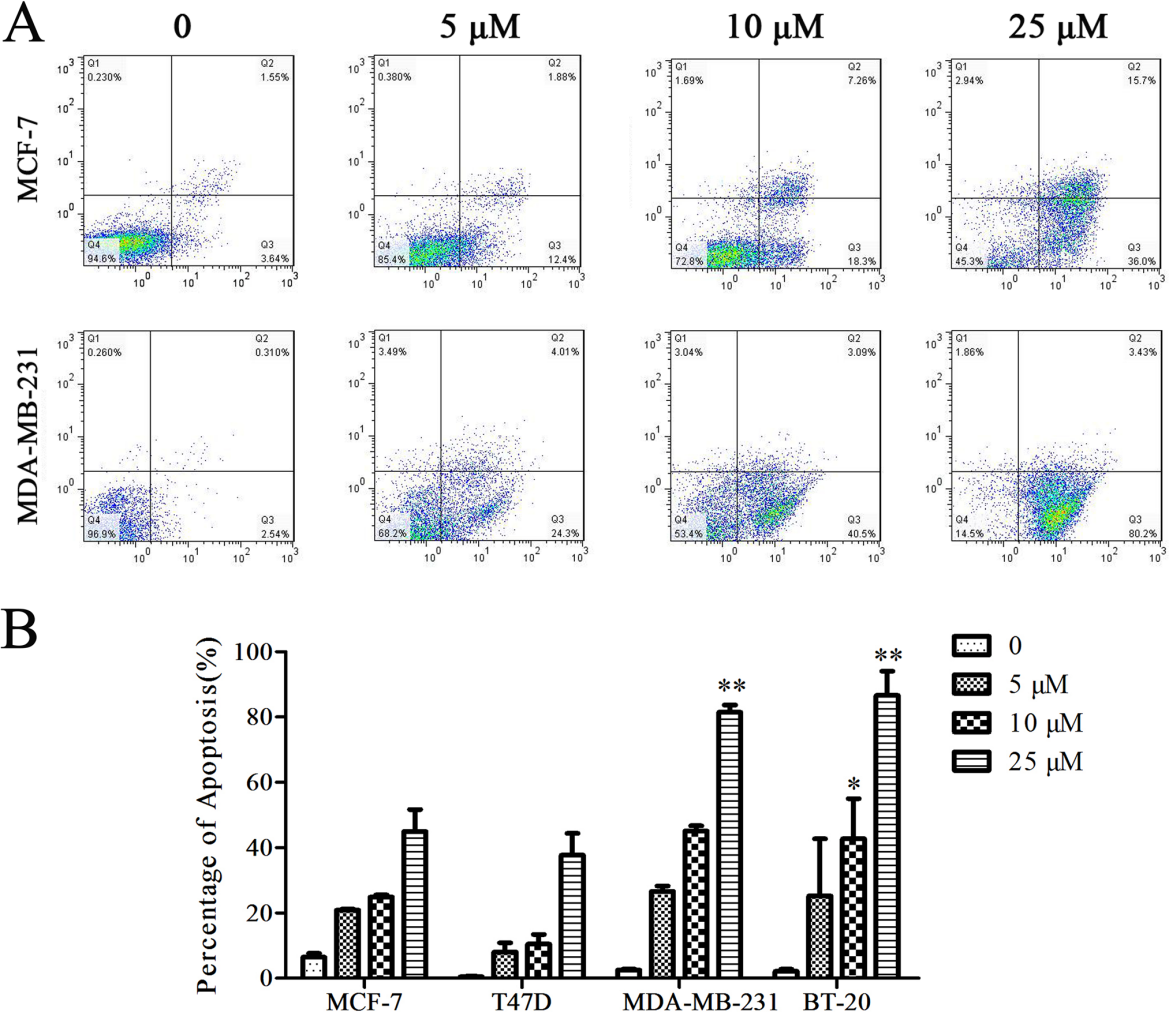
Cu(sal)(phen) induces apoptosis of breast cancer cells MCF-7, T47D, MDA-MB-231 and BT-20 were treated with 0, 5, 10 and 25 μM of Cu(sal)(phen) for 24 hrs, and apoptosis was assessed with the flow cytometry after Annexin V/PI double staining. (**A**). Representative flow cytometry histograms of MCF-7 cells and MDA-MB-231 cells treated with Cu(sal)(phen) or vehicle (DMSO). (**B**). Summary of flow cytometry analyses. Results are represented as the average of two independent experiments. Bars represent mean ± SD. *p < 0.05, **p < 0.01.

To determine the involvement of apoptosis in the Cu(sal)(phen) activity, all breast cancer cell lines MCF-7, T47D, MDA-MB-231 and BT-20 were treated with 5, 10 and 25 μM of Cu(sal)(phen) for 24 hrs, and then apoptosis was analyzed by flow cytometry after Annexin V/PI double staining. The proportion of apoptotic cells upon Cu(sal)(phen) treatment increased in a dose-dependent manner (Figure 2A). MDA-MB-231 TNBC cells showed more cells in early apoptosis compared with MCF-7 non-TNBC cells. Overall, the MDA-MB-231 and BT-20 TNBC cells were more sensitive to Cu(sal)(phen) treatment compared to MCF-7 and T47D non-TNBC cells (Figure 2B). Treatment with 25 μM Cu(sal)(phen) for 24 hrs resulted in more than 80% of TNBC cells (MDA-MB-231 and BT-20) undergoing apoptosis, but only 40% of non-TNBC cells (MCF-7 and T47D). These results indicated that Cu(sal)(phen) induced potent apoptotic activity in TNBC cells.

### Cu(sal)(phen) induces apoptosis through down-regulation of anti-apoptosis proteins in breast cancer cells

**Figure 3:**
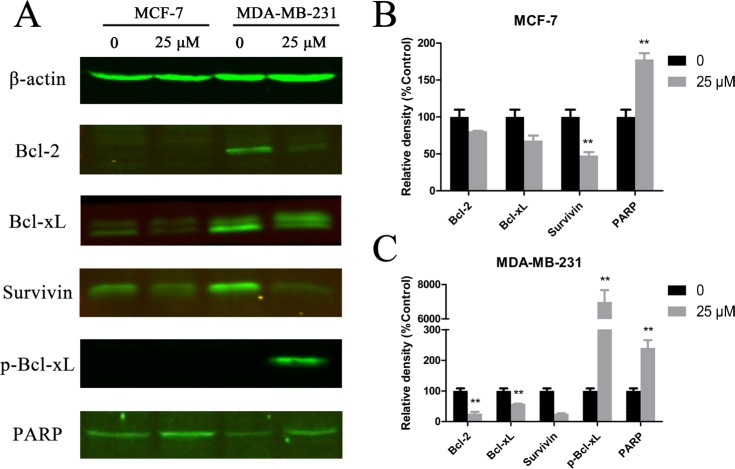
Cu(sal)(phen) treatment down-regulates expression of anti-apoptotic proteins MCF-7 and MDA-MB-231 cells were treated with 25 μM of Cu(sal)(phen) or DMSO as a control and expression of anti-apoptotic proteins was analyzed with Western blot analysis. Actin was used as a loading control. (**A**). Representative Western blot images of Bcl-2, Bcl-xL, phospho-Bcl-xL, survivin and PARP in MCF-7 and MDA-MB-231 cells. (**B**). Quantitative analysis of Western blot images from MCF-7 cells. The densities of target bands were scanned, and values were normalized with actin. (**C**). Quantitative analysis of Western blot images of MDA-MB-231. The densities of target bands were scanned, and values were normalized with actin. Results are represented as the average of two independent experiments. Bars represent mean ± SD. *p < 0.05, **p < 0.01.

To understand the molecular mechanism by which Cu(sal)(phen) induces apoptosis of breast cancer cells, we analyzed the expression levels of several apoptosis-related proteins after drug treatment of MCF-7 and MDA-MB-231 cells by Western blot. As shown in Figure 3, the basal expression levels of Bcl-2, Bcl-xL and survivin were much higher in the MDA-MB-231 TNBC cells than in non-TNBC MCF-7 cells. The expression levels of Bcl-2 and Bcl-xL decreased significantly in MDA-MB-231 cells after drug treatment (Figure 3A). The expression level of survivin was also reduced in MCF-7 and MDA-MB-231 cells treated with 25 μM Cu(sal)(phen) (Figure 3A). Quantification of the Western blot results showed that there was a ~80% decrease in survivin in treated MDA-MB-231 cells compared with a ~43% decrease in MCF-7 cells (Figure 3B). In addition to the reduction of Bcl-xL expression in MDA-MB-231 cells, we also observed a shift of the band, likely corresponding to a change in phosphorylation state of the protein [25]. Indeed, phosphorylation level of Bcl-xL was dramatically increased in MDA-MB-231 cells after the treatment (Figure 3A, 3B). These findings suggest that Cu(sal)(phen) induces apoptosis through down-regulation of anti-apoptosis proteins in breast cancer cells, especially TNBC cells. Moreover, we analyzed the expression of cleaved PARP, which is a marker of activation of apoptosis pathways. We found that the expression of cleaved PARP was increased in both MCF-7 and MDA-MB-231 cell lines after Cu(sal)(phen) treatment (Figure 3A, 3B).

### Cu(sal)(phen) inhibits tumor growth *in vivo*

**Figure 4:**
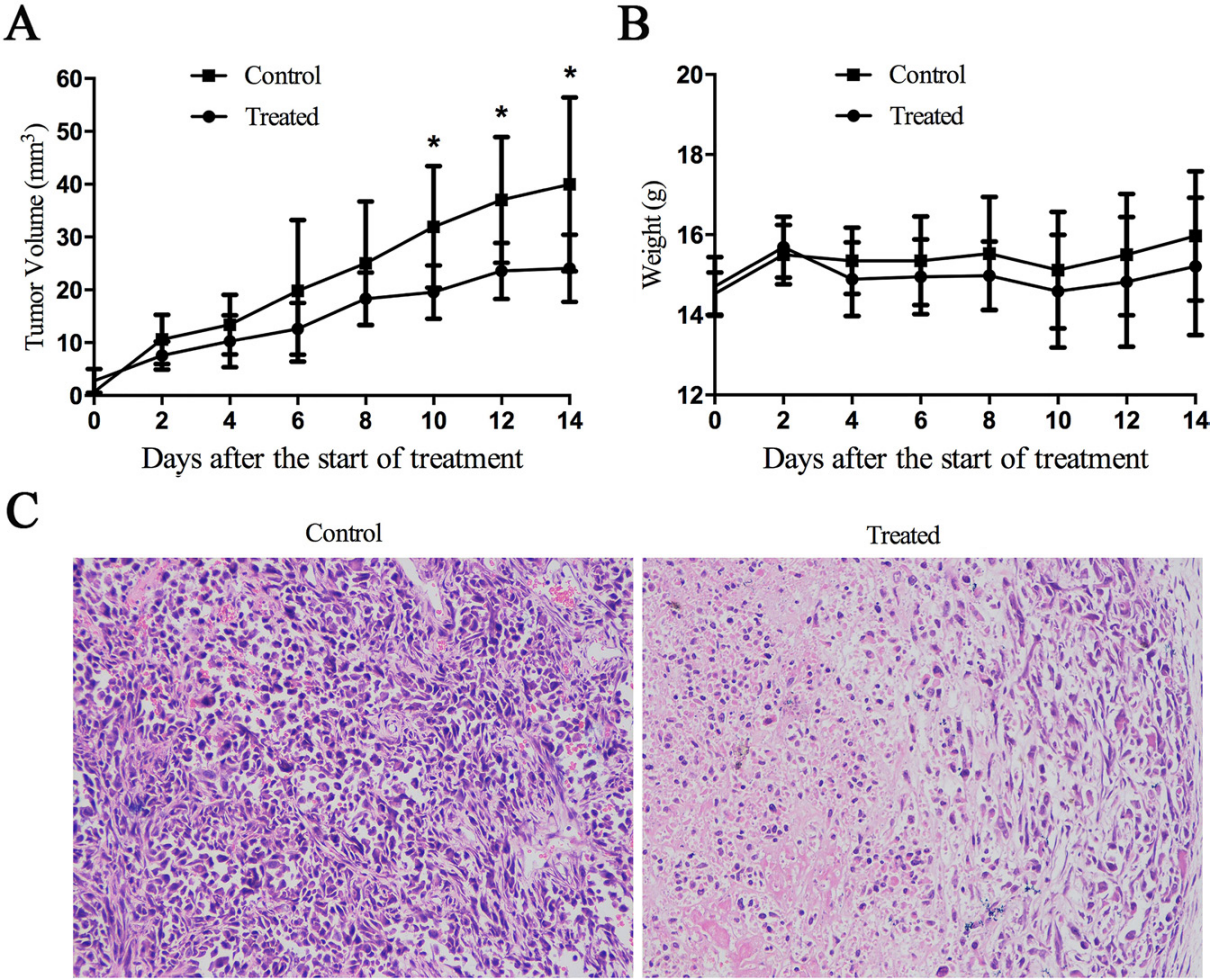
Cu(sal)(phen) suppresses tumor growth *in vivo* 1-mm^3^ tumor block from MDA-MB-231 cells innoculated nude mice was seeded into the mammary fat pad of BALB/c-nu female mice. The mice were injected intraperitoneally (i.p.) every 2 days with 0.1 ml of DMSO or Cu(sal)(phen) (0.5 mg/ml). The tumor size and the body weight were measured every other day throughout the experiments. After 14 days of treatment, the mice were euthanized, the tumors were fixed, sectioned and stained with hematoxylin and eosin (H&E). (**A**). Tumor volume for tumor-bearing mice is shown with mean per group (*n* = 8). *p < 0.05 between the treated and control groups. (**B**). There was no statistical difference of mean body weight between the treated and control groups. (**C**). H&E staining of tumor specimens from the mice injected with Cu(sal)(phen) or DMSO. Magnification, X200.

Breast tumor xenografts were generated in nude mice injected with cultured MDA-MB-231 cells, and then tumor blocks were seeded for second-generation tumor formation. Compared with directly injecting cultured cells, we found that the method of tissue block insertion has many technological advantages, such as higher tumor formation rates, faster tumor growth and more uniform tumor size. Figure 4A shows the tumor growth curves of the two groups; the tumor volume of drug-treated group was significantly reduced compared to the control group at 10 days after drug treatment. Examination of the histopathology of the tumors revealed obvious necrotic areas in tumors from the drug-treated group that were not observed in the control tumors (Figure 4C). Consistent with the drug being well-tolerated, the weight of treated animals was not statistically different from the control animals (Figure 4B). Collectively, these data indicate that, at the concentration used, Cu(sal)(phen) inhibits tumor growth and is well tolerated *in vivo*.

### Cu(sal)(phen) down regulates anti-apoptosis proteins *in vivo*

**Figure 5:**
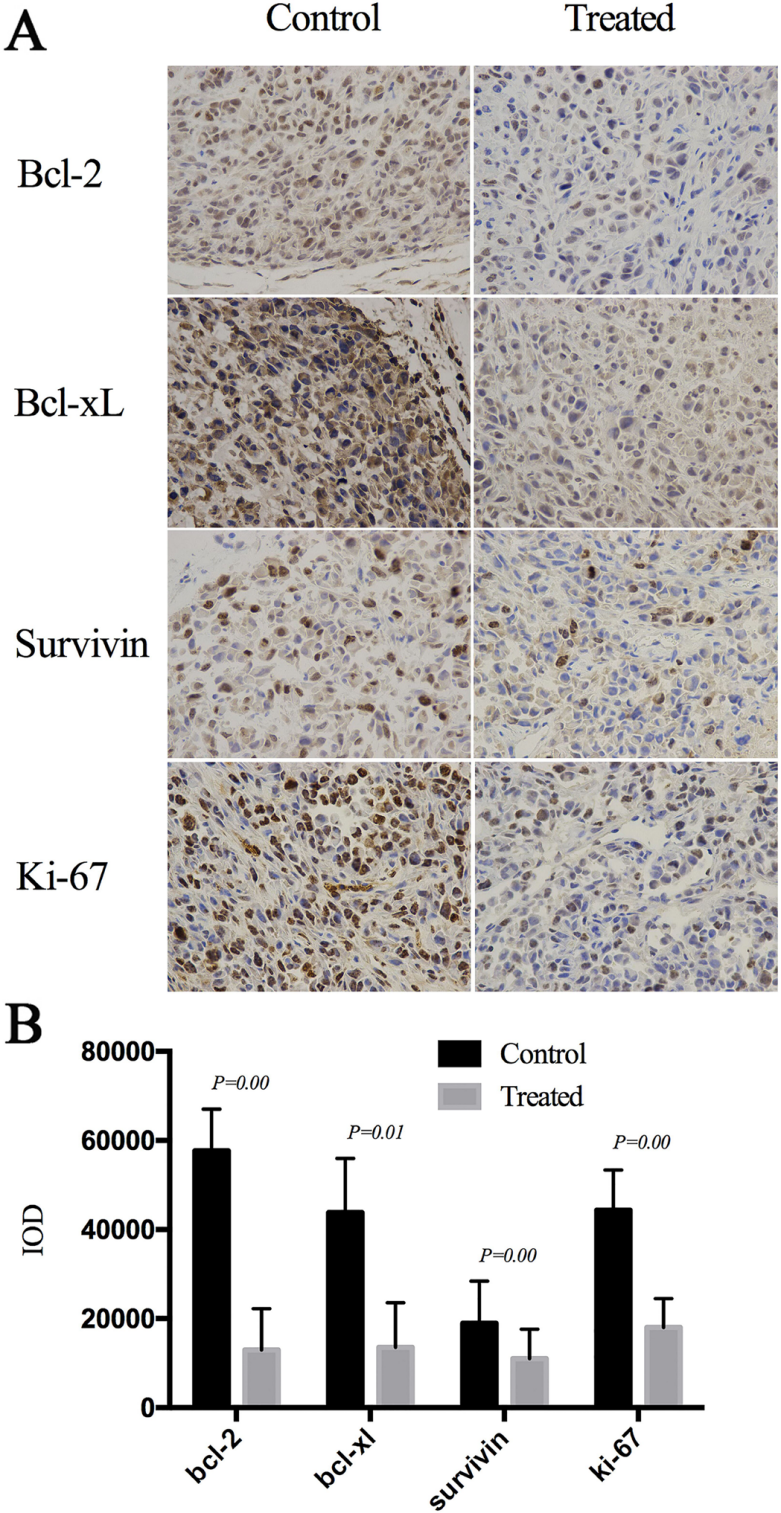
Cu(sal)(phen) down-regulates expression of anti-apoptotic and proliferation-related proteins in xenografted tumors Formalin-fixed, paraffin-embedded sections were stained with antibodies against Bcl-2, Bcl-xL, survivin and Ki-67. Images were taken and the integrated optical density (IOD, *n* = 24 for each protein) was measured by image-pro plus 6.0 software. (**A**). Expression of Bcl-2, Bcl-xL, Survivin and Ki-67 in the treated tumor and control analyzed with Immunohistochemistry. (**B**). Results of semi-quantitative image analysis of immunohistochemistry. The expression levels of all four proteins in the treated and control groups.

We next explored the potential mechanism underlying the anti-tumor activity of Cu(sal)(phen) *in vivo* by examining the expression levels of the apoptotic proteins Bcl-2, Bcl-xL, survivin and proliferation marker Ki-67 by immunohistochemical staining of the tumors and semi-quantitative image analysis. The expression levels of Bcl-2, Bcl-xL, survivin and Ki-67 were all reduced in Cu(sal)(phen)-treated tumors compared to the tumors from the control mice (Figure 5A). The average of integrated optical density (IOD) values of Bcl-2, Bcl-xL, survivin and Ki-67 in the Cu(sal)(phen) treated group was significantly lower than those in the control group (Figure 5B). These results demonstrated that Cu(sal)(phen) down-regulates the expression levels of Bcl-2, Bcl-xL, survivin and Ki-67 in tumors.

## DISCUSSION

It has been shown that Cu(sal)(phen) effectively inhibits growth of cisplatin-sensitive cell lines, as well as cisplatin-resistant cancer cell line [21]. However, the precise molecular mechanism of action has not been well established. In the current study, we demonstrated that Cu(sal)(phen) efficiently induced apoptosis of breast cancer cells, especially TNBC cells. Furthermore, Cu(sal)(phen) inhibited TNBC tumor growth in a xenograft model and induced apoptosis through down-regulating expression of anti-apoptosis proteins Bcl-2, Bcl-xL and survivin both *in vitro* and *in vivo*.

In contrast to the observation that Bcl-2/Bcl-xL were primarily expressed in ER-positive breast cancer cells [26], we found that MDA-MB-231 TNBC cells exhibited much higher level of Bcl-2 and Bcl-xL compared to ER-positive MCF-7 breast cancer cells. Upon Cu(sal)(phen) treatment, the protein levels of Bcl-2 and Bcl-xL were dramatically down-regulated in MDA-MB-231 cells. In addition, the level of the phosphorylated Bcl-xL was significantly increased in MDA-MB-231 cells. Bcl-xL is phosphorylated at the activation loop on the Ser62 by JNK, which modulates the Bax/Bcl-xL interactions and inhibits Bcl-xL’s anti-apoptotic activity [25, 27]. These data suggest that multiple mechanisms are likely to be involved in the induction of apoptosis by Cu(sal)(phen).

Survivin primarily inhibits apoptosis through suppressing activation of caspase-3 and caspase-7, interfering with caspase-9 activity, and blocking the downstream effectors of apoptosis pathways [28]. Survivin is often overexpressed in breast cancer [29], and is associated with less apoptosis [30], poor overall survival [31], and resistance to neoadjuvant chemotherapy [32]. Several therapeutic approaches targeting survivin have been tested, including inhibition of *survivin* transcription, post-translational inhibition of survivin, vaccines against survivin, and gene therapy approaches such as gene editing [33]. In this study, we found that the expression of survivin protein was dramatically reduced by Cu(sal)(phen) treatment in breast cancer cells, especially in TNBC cells, suggesting that Cu(sal)(phen) may provide a promising approach to target survivin expression.

In this study, we also found that Cu(sal)(phen) treatment attenuated tumor growth *in vivo*. Similar to what was observed *in vitro*, the expression levels of Bcl-2, Bcl-xL and survivin proteins were down-regulated in tumor tissue from drug-treated animals as detected by immunohistochemistry. These findings suggest that apoptosis induced by Cu(sal)(phen) is likely to be responsible for its anti-tumor activity. Interestingly, Ki67, a proliferation marker of tumor [34] and a prognosis marker for breast cancer, was also down-regulated in tumors in animals treated with Cu(sal)(phen), suggesting that Cu(sal)(phen) treatment also suppressed tumor cell proliferation.

Although targeted therapies have long been sought for TNBC, there has been limited success. Interestingly, we found that the growth inhibitory activity of Cu(sal)(phen) was more profound in TNBC cell lines, compared to ER-positive MCF-7 cells. One of the concerns for developing Cu(sal)(phen) as a therapeutic agent for TNBC treatment is toxicity. Although our *in vivo* experiment showed that mice well tolerated the dose that effectively suppressed the tumor growth, additional experiments are required in order to further develop it into an effective, less toxic, novel therapeutic agent for human TNBC.

## MATERIALS AND METHODS

### Chemicals

Cu(sal)(phen) was synthesized by Dinglong Chemicals (Wuhan, Hubei, China), with a two-step reaction. In the first step, 9.7 g of copper hydroxide Cu(OH)_2_ and 56.2 g of salicylic acid were refluxed in 600 ml pure ethanol for 8 hrs. Upon cooling to room temperature, a yellowish precipitate was collected by filtration. The solid product was further purified by ethanol for three times, and dried in vacuum at 60°C to generate Cu(sal)_2_(H_2_O)_2_. In the second step, 3.8 g of the product from the step 1 and 4.3 g of 1,10-phenanthroline were refluxed in 350 ml pure ethanol for 4 hrs, to generate a greenish mixture. After cooled to room temperature, the green precipitate was collected by filtration, and washed by hot ethanol for three times. The final product Cu(sal)(phen) was dried at 75°C in a vacuum for 8 hrs. 5mM stock solution was made by dissolving Cu(sal)(phen) in dimethyl sulfoxide (DMSO) and diluted when used. The solvent DMSO was used as mock control.

### Cell lines

MCF-7, T47D, MDA-MB-231 and BT-20 cell lines were obtained from the American Type Culture Collection (Rockville, MD, USA). MCF-7 and T47D cells were cultured in DMEM (Fisher Scientific, Hanover Park, IL). MDA-MB-231 and BT-20 cells were maintained in RPMI 1640 (Invitrogen, Grand Island, NY, USA). The media were supplemented with 10% FBS and 1% penicillin/streptomycin. Cell lines under 20 passages were used for all experiments.

### Cell growth assays

Cell growth was analyzed with the MTS assay using CellTiter 96^®^ AQueous One Solution Cell Proliferation Assay (Promega, WI, USA) according to the manufacture recommendation. Briefly, cells were seeded in 96-well culture plates. Following treatment with Cu(sal)(phen) or DMSO as a control, cell viability was assessed by adding 3-(4,5-dimethylthiazol-2-yl)-5-(3-carboxymethoxyphenyl)-2-(4-sulfophenyl)-2H-tetrazolium (MTS) to the culture medium and incubated for 4 hrs at 37°C. The optical density was measured at 490 nm using a Synergy 2 plate reader (Biotek, VT, USA).

### Apoptosis assay

Apoptosis was assessed quantitatively using Annexin V (BD Biosciences) and propidium iodide (PI) double staining. After treatment, cells were harvested by trypsinization, stained with Annexin V-FITC, and 50 μg/mL of PI solution, then immediately analyzed by the Coulter Epics XL Flow Cytometer (Beckman, USA). Experiments were performed in triplicate, and a total of 10,000 cells were analyzed in each experiment.

### Western blotting

Total cell lysate was extracted in the RIPA buffer containing protease inhibitor cocktail as described previously [35]. 25 μg of lysate from each cell line was separated using SDS-PAGE gel and transferred to Immobilon P membrane (Millipore). Blots were probed with the following antibodies: anti-Bcl-2 antibody (2870, Cell Signaling, USA), anti-Bcl-xL (2764, Cell Signaling, USA), anti-phospho-Bcl-xL (sc-101644, Santa Cruz, USA), anti-survivin (2808, Cell Signaling, USA) and anti-actin monoclonal antibody (Sigma, USA) in blocking buffer (Li-Cor, NB, USA). Then the membranes were incubated with IRDye secondary antibodies (Licor, NB, USA). The membranes were scanned and the images were captured with Odyssey SA (Licor, NB, USA). Images were analyzed and quantified using ImageStudio software.

### Xenograft transplantation assays

All animal studies were performed in accordance with the Novartis Institutes for BioMedical Research (NIBR) Animal Care and Use Committee protocols and regulations approved by Institutional Animal Care and Use Committee of Wuhan University. Four-week-old BALB/c-nu female mice were purchased from Hunan SJA Laboratory Animal Co. Ltd. (Hunan, China), and tumors were generated by subcutaneous injection of 5 × 10^6^ MDA-MB-231 cells as described previously [36]. When the diameters of the tumors reached 1 cm, the tumors were removed and cut into 1-mm^3^ tumor blocks. The tumor blocks were seeded for the second-generation tumors. After implantation in subcutaneous tissue, the mice were divided randomly into two groups of eight mice each. The tumor diameter was measured every other day, and tumor volume was calculated as described previously [37]. When the diameter of the tumor was greater than 3mm, animals were treated with either vehicle (DMSO) or Cu(sal)(phen) every other day by intraperitoneally (i.p.) injection with 100 μl of DMSO or 0.5mg/ml Cu(sal)(phen) in the same volume of DMSO, as determined by a pilot experiment. Animal body weights were monitored every other day. After 14 days of treatment, the mice were euthanized, the tumors were fixed in paraformaldehyde, embedded in paraffin, sectioned and stained with hematoxylin and eosin (H&E).

### Immunohistochemistry assays

Immunohistochemistry was performed on formalin-fixed, paraffin-embedded sections with the following antibodies: anti-Bcl-2 (ab28819,Abcam), anti-Bcl-xL (ab180849, Abcam), anti-survivin (ab8228, Abcam) and anti-Ki-67 (ab6526, Abcam). Antigen retrieval was carried out in boiling EDTA buffer. The biotinylated secondary antibody was added to the sections. Finally, tissue sections were stained with DAB solution. Three areas of each tumor tissue section were randomly selected, and images were taken at a 400-fold magnification (Olympus BX51). Positive cells on three sections for each tumor were quantified. The integrated optical density (IOD, *n* = 24) was measured by Image-pro plus 6.0 software.

### Statistical analysis

All experiments were performed at least twice, and statistical analyses were performed using a one-way ANOVA with a Student’s *t*-test post-test, unless noted otherwise. For quantification, means are shown with standard deviation (SD). For immunohistochemistry analysis, the comparison between the experimental group and the control group was assessed by *t*-test and performed with SPSS 16.0 software. A value of *p* < 0.05 was considered statistically significant.

## Abbreviations

Cu(sal)(phen): Salicylate ●Phenanthroline Copper (II) Complex
DMSO: Dimethyl sulfoxide
FBS: fetal bovine serum
H&E: hematoxylin and eosin
HER-2: human epidermal growth factor receptor 2
IAP: inhibitor of apoptosis proteins
IOD: integrated optical density
MTS: 3-(4,5-dimethylthiazol-2-yl)-5-(3-carboxymethoxyphenyl)-2-(4-sulfophenyl)-2H-tetrazolium
OS: overall survival
PARP: poly ADP-ribose polymerase
pCR: pathologic complete response
PI: propidium iodide
SOD: superoxide dismutase
TNBC: Triple-negative breast cancer.
